# Prescription of EPs 7630 is associated with short- and long-term benefits in acute bronchitis: a real-world data analysis

**DOI:** 10.3389/fphar.2025.1652203

**Published:** 2025-09-15

**Authors:** Adrian Gillissen, Thorsten Reineke, Martin Burkart, Petra Funk, Charles Christian Adarkwah, Karel Kostev

**Affiliations:** ^1^ Department of Pulmonary and Intensive Care Medicine, Klinikum Stuttgart, Katharinenhospital, Stuttgart, Germany; ^2^ Research and Development, Dr. Willmar Schwabe GmbH & Co. KG, Karlsruhe, Germany; ^3^ Global Medical Affairs, Dr. Schwabe Holding SE & Co. KG, Karlsruhe, Germany; ^4^ Institute of General Practice, Philipps-University Marburg, Marburg, Germany; ^5^ Epidemiology, IQVIA, Frankfurt a. M., Germany

**Keywords:** EPs^®^ 7630, phytopharmaceuticals, acute bronchitis, cough, antibiotics, ambroxol, acetylcysteine, population-based analysis

## Abstract

**Introduction:**

Acute bronchitis is caused by an infection of the lower respiratory tract, resulting in considerable distress of patients and substantial economic costs due to lost workdays. Although acute bronchitis is mostly caused by viruses, antibiotics are frequently used. The aim of this study was to evaluate whether a prescription of EPs 7630 for patients with acute bronchitis is associated with a lower recurrence rate of the disease or fewer complications, a reduced need for antibiotic therapy, or fewer lost workdays.

**Methods:**

A retrospective analysis was conducted using the IQVIA™ Disease Analyzer database, which contains information from nearly 3,000 office-based physicians, representing around 3%–5% of all German practices. We analyzed the rates of recurrence of acute bronchitis, antibiotic prescription, duration of sick leave, and incidence of complications (pneumonia, chronic bronchitis) with regard to their association with prescriptions of EPs 7630, ambroxol, acetylcysteine, or antibiotics for acute bronchitis.

**Results:**

Of the 376,366 patients suffering from acute bronchitis between January 2005 and December 2022, 1,994 received prescription of EPs 7630, 14,952 ambroxol, 24,149 acetylcysteine, and 335,271 antibiotics. EPs 7630 prescription was associated with a significantly lower likelihood of re-consultation due to acute bronchitis within 365 days compared to ambroxol (HR: 0.74; 95% CI: 0.65–0.83), acetylcysteine (HR: 0.78; 95% CI: 0.69–0.88), and antibiotics (HR: 0.75; 95% CI: 0.67–0.85) (p < 0.001 each). The incidence of an antibiotic prescription following an initial EPs 7630 prescription was significantly lower compared to acetylcysteine (HR: 0.89; 95% CI: 0.81–0.98) (p = 0.020). EPs 7630 was also associated with a lower risk of pneumonia and chronic bronchitis and a lower proportion of patients with sick leave lasting >3 and ≥7 days, respectively, compared to the other prescription groups.

**Conclusion:**

Prescribing EPs 7630 is associated with a lower incidence of disease recurrence and complications, a reduced need for antibiotic therapy, and a reduction in lost workdays. The results of this retrospective analysis thus emphasize the overall benefits of EPs 7630 as an effective treatment option for managing acute bronchitis.

## 1 Introduction

Acute bronchitis is a widespread disease, affecting an estimated 5% of the adult population each year, with a large proportion of those affected seeking medical help (Braman, 2006; Kleibrink and Taube, 2019). As reported from an observational study in twelve European countries, acute bronchitis is one of the main reasons why patients are absent from work or school, with an average absence of 4 days (Hordijk et al., 2015). The disease is characterized by an acute cough, with or without sputum production, and signs of a lower respiratory tract infection, affecting the air tubes (bronchi). Acute bronchitis is typically caused by viral infections - common viral pathogens including rhinoviruses, influenza and parainfluenza viruses, and coronaviruses -, while bacteria are detected in only 1%–10% of cases (Kinkade and Long, 2016). Acute bronchitis usually resolves spontaneously (Wark, 2015). In some cases, however, the disease can lead to a chronic bronchitis and may be a precursor to a community-acquired pneumonia (CAP). Risk factors for complications include smoking, a pre-existing lung condition such as the chronic obstructive pulmonary disease or bronchiectasis, or a disorder of the immune system that predisposes to CAP, for example (Wark, 2015).

Treatment for acute bronchitis is usually supportive, focusing on relieving symptoms. Mucolytic drugs, such as ambroxol or acetylcysteine (Kleibrink and Taube, 2019), or phytopharmaceutical formulations (Soilemezi et al., 2020) are the cornerstone of these treatment efforts. Antibiotics are generally not recommended unless a bacterial infection is present, causing a treatable disorder. Despite this evidence-based recommendation, antibiotics are often prescribed in primary care (Kern and Kostev, 2021). The motivation for this phenomenon is attributable to a number of factors, including the physician’s intention to provide rapid symptom relief, the perceived pressure from patients, and the assumption of a possible bacterial superinfection despite a lack of scientific evidence (Schubert et al., 2023; Wollny et al., 2023). Just to the contrary, unnecessary antibiotic use contributes to antibiotic resistance and must therefore be avoided (Ventola, 2015). However, the rate of prescribing antimicrobials and their inappropriate use remain high and vary around the world (Kasse et al., 2024).

The prescription rate for antibiotics in Germany is low compared to other European countries (ECDC, 2020). However, there are regional differences, and the target rate of prescription of fewer than 20% is not always met (Arens et al., 2024). A retrospective study analyzing data from German pediatric practices showed that the antibiotic prescriptions for acute bronchitis and other acute respiratory infections had decreased, while the prescription of phytopharmaceuticals had increased over the last decade (Kostev et al., 2023). Real-world data also indicate that treating acute respiratory infections with phytopharmaceuticals may contribute to fewer antibiotic prescriptions and shorter periods of sick leave (Martin et al., 2020). These results underline the importance of phytotherapeutic approaches in treating acute respiratory tract infections, particularly acute bronchitis.

A particularly interesting phytopharmaceutical option for the treatment of acute bronchitis is EPs 7630, an extract from the roots of *Pelargonium sidoides* with a drug extract ratio of 1:8–10, extraction solvent: ethanol 11% (w/w) (EPs^®^ 7630 is a proprietary extract and active ingredient in pharmaceuticals manufactured by Dr. Willmar Schwabe GmbH & Co. KG). According to the European Pharmacopoeia monograph, the plant material has to contain not less than 2% of tannins. It is composed of carbohydrates, minerals, peptides, purine derivatives, highly substituted benzopyranones, and oligo- and polymeric prodelphinidins (Schoetz et al., 2008; Emanuel et al., 2023).

Several studies have shown that EPs 7630 has anti-inflammatory, antiviral, and immunomodulatory properties (Cinatl et al., 2024). *In vitro* studies, for example, have indicated, in addition to direct antiviral and moderate antibacterial effects, notable immunomodulatory capabilities of EPs 7630 and its isolated constituents, which are in part based on the modulation of the mitogen-activated protein (MAP) kinase pathway which regulates various cytokines such as tumor necrosis factor α, interferon-β, or interleukin-22 (Kolodziej et al., 2003; Witte et al., 2015; 2020). In mice and guinea pigs, oral administration of EPs 7630 at human-equivalent doses resulted in antitussive, secretolytic, and anti-inflammatory effects (Bao et al., 2015). Additionally, EPs 7630 reduces rhinovirus infection in human bronchial cells by modulation of viral binding proteins and interfering with the virus replication (Michaelis et al., 2011; Roth et al., 2019; Roth et al., 2021). EPs 7630 also interacts with the replication of SARS-CoV-2 as well as innate immune responses in the human lung cell line Calu-3, as shown by *in vitro* studies (Papies et al., 2021a; Papies et al., 2021b).

EPs 7630 has been investigated in more than 30 clinical trials for the treatment of acute respiratory tract infections, resulting in extensive evidence of the extract’s efficacy and tolerability (Agbabiaka et al., 2008; 2009; Matthys et al., 2013; Timmer et al., 2013; Matthys et al., 2016; Kamin et al., 2022). A systematic review and meta-analysis of randomized, placebo-controlled clinical trials showed EPs 7630 to be superior to placebo in treating acute bronchitis and in reducing the Bronchitis Severity Scale (BSS) total score during a 7-day treatment period (Matthys et al., 2016). Patients treated with EPs 7630 also perceived a faster onset of treatment effect and showed a higher recovery rate compared to placebo. EPs 7630 was also shown to reduce the duration of symptoms, sick leave, and absence from school (Kardos et al., 2022; Matthys et al., 2023; Zwiauer et al., 2024).

Despite the wealth of research, there is a lack of real-life data to further elucidate the role of EPs 7630 in the treatment of acute bronchitis with respect to 1) whether it is effective in comparison to other drugs with secretolytic and anti-inflammatory properties (including antimicrobial medications) that are usually prescribed for acute bronchitis and to 2) whether it may provide long-term benefits beyond the acute treatment effects demonstrated in randomized clinical trials. A recent real-world cohort study showed that prescribing EPs 7630 was associated with fewer recurrences, antibiotic prescriptions, and complications in acute sinusitis (Tisch et al., 2024). We therefore evaluated whether such long-term benefits also apply to prescription of EPs 7630 in acute bronchitis, focusing on the association with less frequent recurrences of acute bronchitis, fewer antibiotic prescriptions, shorter or less frequent sick leave, a lower incidence of CAP, and a lower occurrence of chronic bronchitis.

## 2 Materials and methods

### 2.1 Study design

This study is a retrospective analysis based on the IQVIA™ Disease Analyzer database. The IQVIA™ Disease Analyzer database contains case information provided by office-based physicians (both general practitioners (GP) and specialists) in Germany. Information is available on patient demographics, drug prescriptions, concomitant medication, comorbid conditions, sick leave, and referrals to hospitals. The database contains data from more than ten million patients documented between 2005 and 2022. Information is provided by nearly 3,000 office-based physicians, representing approximately 3%–5% of all German practices (IMS^®^DA status date: March 2024). The sample is geographically representative for Germany, covering eight major German regions. Since analyses did not indicate any lack of representativeness or validity, the database appears to be suitable for pharmaco-epidemiological and pharmaco-economic studies (Rathmann et al., 2018). The IQVIA™ Disease Analyzer database has already been used for studies of upper respiratory infections in the past (Tanislav and Kostev, 2022; Loosen et al., 2023).

### 2.2 Study population

This study included data from patients aged 18 and over in an outpatient care setting in Germany (i.e., visits to GPs’ offices) for whom a diagnosis of acute bronchitis (ICD-10: J20 excluding J20.0, J20.1, J20.2) has been documented in the IQVIA™ Disease Analyzer database at least once between January 2005 and December 2022. The day of first diagnosis was used as the index date. Patients with a prescription of one of the study medications on the day of diagnosis were categorized into one of four cohorts: 1) EPs 7630, 2) ambroxol, 3) acetylcysteine, and 4) antibiotics. The respective prescribed therapeutic dosage was at the discretion of the attending physician. Patients with a prescription of one of the study medications within 30 days prior to the index date, with prescriptions of other cough medications (ATC: R05 - cough) within 30 days prior to the index date, with prescriptions of more than one study medication on the index date, with a diagnosis of chronic bronchitis (ICD-10: J40-J42) prior to or on the index date, or without documented information on age and sex were excluded.

### 2.3 Statistical analysis

This study analyzed whether the prescription of EPs 7630 shortly after the diagnosis of acute bronchitis was associated with any of the following 5 outcomes, based on the corresponding Anatomical Therapeutic Chemical (ATC) codes and International Classification of Diseases (ICD) codes: (1) less frequent recurrence of acute bronchitis (ICD-10: J20), (2) less frequent antibiotic prescriptions (ATC: J01), (3) less or shorter sick leave, (4) lower incidence of CAP (ICD-10: J12-J18), or (5) lower incidence of chronic bronchitis (ICD-10: J40-42). For each outcome, an independent analysis was performed.

To reduce selection bias and impact of co-variables on the outcomes, a matched-pairs design was used. Patients of the ambroxol, acetylcysteine, and antibiotics cohorts were separately matched to patients with an EPs 7630 prescription (1:5) using a nearest neighbor propensity score based on age, sex, health insurance status, Charlson Comorbidity Index (CCI), and diagnosis of asthma (ICD-10: J45) or COPD (ICD-10: J44) documented within 12 months prior to or on the index date. The CCI is a score system based on 17 comorbidities with a total score that (theoretically) ranges from 0 to 37 (Charlson et al., 1987). Higher scores indicate higher morbidity (Kuss et al., 2016). Standardized mean difference (SMD) was used to examine the balance of covariate distribution between treatment groups. We allowed a SMD of ≤0.1, indicating that an adequate covariate balance had been achieved (Stuart et al., 2013). Kaplan-Meier analyses were conducted to estimate the cumulative incidence of the following outcomes within 365 days after index date: a renewed confirmed diagnosis of acute bronchitis, a first antibiotic prescription, a CAP diagnosis, and a first-time diagnosis of chronic bronchitis. The association between prescription of EPs 7630 and these outcomes was analyzed via Cox regression analyses and compared to ambroxol, acetylcysteine, and antibiotics. Results of the Cox regression were given as Hazard Ratios (HR) with 95% confidence intervals (95% CI) and corresponding p-values. Kaplan-Meier analyses for a renewed confirmed diagnosis of acute bronchitis indicated that the EPs 7630 cohort differed from the other cohorts both in the short term, probably representing the acute episode of acute bronchitis, and for the rest of the 365 days. Therefore, Cox regressions were calculated for two periods in a post-hoc sensitivity analysis: 1–7 days after the index date and 8–365 days after the index date. As patients in the antibiotics cohort had already received an antibiotic as an initial prescription, the Kaplan-Meier analysis and Cox regression versus this cohort for a new antibiotic prescription were only performed for the period of 31–365 days after the index date. For evaluation of sick leave, the duration as recorded by the physician in the database on the day of consultation was used. The proportions of patients with a recorded sick leave of ≥1 day associated with acute bronchitis were compared between study cohorts using logistic regression analyses. Different sick leave durations (more than 3, at least 7, or at least 14 days, respectively) were included as dependent variable, and pharmaceutical therapy (EPs 7630 versus ambroxol, acetylcysteine, antibiotics) as independent variable. Results of the logistic regression were displayed as Odds Ratios (OR) with 95% CI. Additionally, sick leave analysis was repeated for patients with recorded sick leave compared to patients without recorded sick leave.

## 3 Results

**FIGURE 1:**
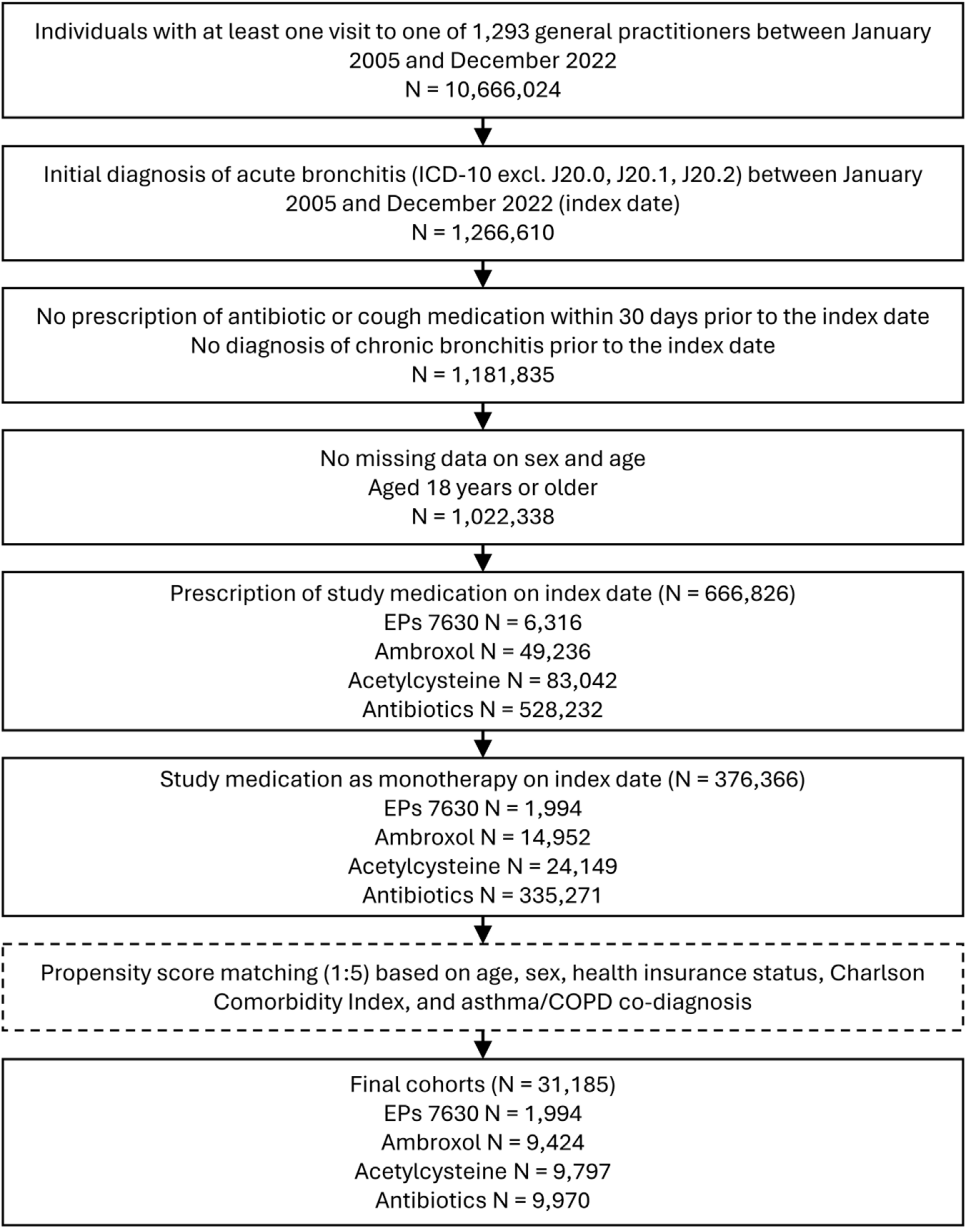
Selection of study participants.

Of 376,366 patients fulfilling all inclusion criteria, EPs 7630 was prescribed to 1,994, ambroxol to 14,952, acetylcysteine to 24,149, and antibiotics to 335,271 patients (Figure 1).

Selection of study participants resulted in final propensity-matched cohorts including 1,994 patients in the EPs 7630 cohort, 9,424 patients in the ambroxol cohort, 9,797 patients in the acetylcysteine cohort, and 9,970 patients in the antibiotics cohort. Baseline characteristics of the study participants are shown in Table 1. Matching was restricted by differences in the proportion of privately insured patients between cohorts. The final study cohorts were well matched in terms of age, sex, private health insurance coverage, diagnosis of asthma or COPD, or CCI. In the antibiotics cohort, the most commonly prescribed antibiotics were amoxicillin (17.8% of patients), azithromycin (16.1%), cefuroxime axetil (12.5%), clarithromycin (10.6%), roxithromycin (9.3%), doxycycline (8.2%), levofloxacin (5.0%), ciprofloxacin (4.7%), amoxicillin/clavulanic acid (3.4%), and moxifloxacin (2.5%).

**TABLE 1:** Basic characteristics of study participants.

Variable	EPs 7630	Ambroxol	Acetylcysteine	Antibiotics
N	1,994	9,424	9,797	9,970
Female sex (n, %)	1,072 (53.8)	5,036 (53.4)	5,170 (52.8)	5,362 (53.8)
Age (mean, SD)	43.2 (16.9)	43.7 (18.0)	43.4 (17.2)	43.2 (16.9)
18–30 years (n, %)	567 (28.4)	2,786 (29.6)	2,782 (28.4)	2,836 (28.4)
31–45 years (n, %)	390 (29.6)	2,605 (27.6)	2,883 (29.4)	2,941 (29.5)
46–65 years (n, %)	604 (30.3)	2,710 (28.8)	2,921 (29.8)	3,016 (30.3)
> 65 years (n, %)	233 (11.7)	1,323 (14.0)	1,211 (12.4)	1,177 (11.8)
Private health insurance coverage (n, %)	300 (15.1)	954 (10.1)	1,327 (13.5)	1,500 (15.1)
CCI (median, IQR)	0 (0–1)	0 (0–1)	0 (0–1)	0 (0–1)
Asthma/COPD* (n, %)	175 (8.8)	701 (7.4)	770 (7.9)	866 (8.7)
Asthma	115 (5.8)	470 (5.0)	531 (5.4)	615 (6.2)
COPD	73 (3.7)	314 (3.3)	320 (3.3)	357 (3.6)

CCI, Charlson comorbidity index; COPD, chronic obstructive pulmonary disease; IQR, interquartile range; SD, standard deviation.

*This includes patients with a diagnosis of asthma or COPD, as well as patients with a diagnosis of both. Therefore, the sum does not correspond to the numbers of the separate analysis of patients with a diagnosis of asthma or COPD (below).

### 3.1 Recurrence of acute bronchitis

**FIGURE 2:**
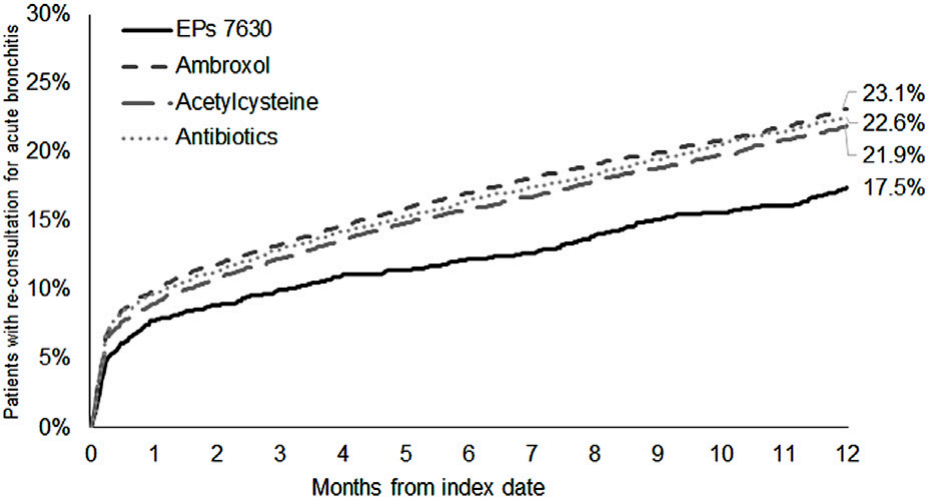
Cumulative incidence of renewed acute bronchitis diagnosis in the period 1–365 days after the index date.

17.5% of patients with a prescription of EPs 7630 and ≥21.9% of patients in the respective other cohorts had a re-consultation for acute bronchitis within 365 days after the index date (Figure 2). EPs 7630 prescription was associated with a significantly lower incidence of a renewed confirmed diagnosis of acute bronchitis compared to ambroxol (HR: 0.74; 95% CI: 0.65–0.83), acetylcysteine (HR: 0.78; 95% CI: 0.69–0.88), and antibiotics (HR: 0.75; 95% CI: 0.67–0.85) within 365 days after the index date (p < 0.001 each; Table 2). In both periods, 1–7 days after the index date and 8–365 days after the index date, EPs 7630 prescription was associated with a significantly lower incidence of re-consultation for acute bronchitis compared to the ambroxol (p = 0.001 and p < 0.001, respectively), acetylcysteine (p = 0.012 and p = 0.001, respectively), and antibiotics cohorts (p = 0.001 and p < 0.001, respectively) (Table 3).

**TABLE 2:** Associations between EPs 7630 prescription and renewed confirmed diagnosis of acute bronchitis, antibiotic prescription, chronic bronchitis, and CAP 1–365 days after the index date (Cox regression models).

	HR (95% CI)	P-value
Renewed confirmed diagnosis of acute bronchitis		
EPs 7630 vs ambroxol	0.74 (0.65–0.83)	<0.001
EPs 7630 vs acetylcysteine	0.78 (0.69–0.88)	<0.001
EPs 7630 vs antibiotics	0.75 (0.67–0.85)	<0.001
Antibiotic prescription		
EPs 7630 vs ambroxol	0.93 (0.84–1.02)	0.112
EPs 7630 vs acetylcysteine	0.89 (0.81–0.98)	0.020
EPs 7630 vs antibiotics*	0.56 (0.50–0.63)	<0.001
Chronic bronchitis		
EPs 7630 vs ambroxol	0.78 (0.70–0.88)	<0.001
EPs 7630 vs acetylcysteine	0.81 (0.72–0.91)	<0.001
EPs 7630 vs antibiotics	0.94 (0.83–1.05)	0.261
CAP		
EPs 7630 vs ambroxol	0.66 (0.39–1.11)	0.13
EPs 7630 vs acetylcysteine	0.49 (0.29–0.82)	<0.003
EPs 7630 vs antibiotics	0.48 (0.29–0.80)	0.005

CAP, community-acquired pneumonia; CI, confidence interval; HR, hazard ratio; *due to the initial antibiotic prescription, only new antibiotic prescriptions from day 31 after the index date were considered.

**TABLE 3:** Association between EPs 7630 prescription and renewed confirmed diagnosis of acute bronchitis 1 to 7 and 8–356 days after the index date (Cox regression models).

	1–7 days after the index date		8–356 days after the index date	
	HR (95% CI)	P-value	HR (95% CI)	P-value
EPs 7630 vs ambroxol	0.69 (0.55–0.86)	0.001	0.75 (0.65–0.86)	<0.001
EPs 7630 vs acetylcysteine	0.74 (0.59–0.94)	0.012	0.79 (0.68–0.91)	0.001
EPs 7630 vs antibiotics	0.68 (0.54–0.86)	0.001	0.77 (0.67–0.89)	<0.001

CI, confidence interval; HR, hazard ratio.

### 3.2 New antibiotic prescription

**FIGURE 3:**
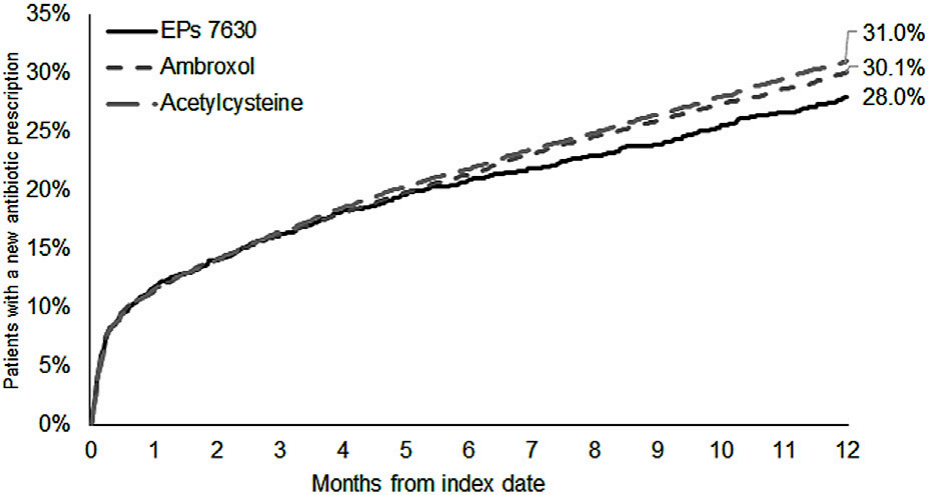
Cumulative incidence of antibiotic prescription in the period 1–365 days after the index date (EPs 7630, ambroxol, and acetylcysteine cohorts).

**FIGURE 4:**
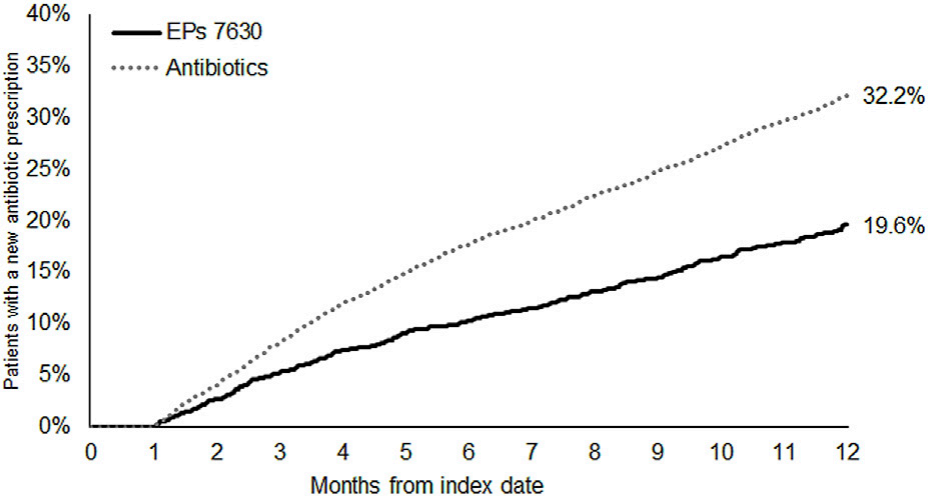
Cumulative incidence of antibiotic prescription in the period 31–365 days after the index date (EPs 7630 and antibiotics cohorts).

The cumulative incidence of a new antibiotic prescription was slightly lower in the EPs 7630 cohort (28.0%) compared to the ambroxol (30.1%) and acetylcysteine (31.0%) cohorts (Figure 3). EPs 7,630 prescription was associated with a significantly lower incidence of antibiotic prescription within 365 days after the index date compared to acetylcysteine (HR: 0.89; 95% CI: 0.81–0.98) (p = 0.020; Table 2). There was no significant difference compared to the ambroxol cohort (HR: 0.93; 95% CI: 0.84–1.02) (Table 2). As all patients in the antibiotics cohort already received an antibiotic as an initial prescription, only the period 31–365 days after the index days was analyzed for comparison of this cohort with the EPs 7630 cohort. Analysis showed that EPs 7630 prescription was associated with a significantly lower incidence of an antibiotic prescription in the period 31–365 days after the index date compared to the antibiotics group (HR: 0.56; 95% CI: 0.50–0.63) (p < 0.001; Table 2; Figure 4).

### 3.3 Incidence of chronic bronchitis

**FIGURE 5:**
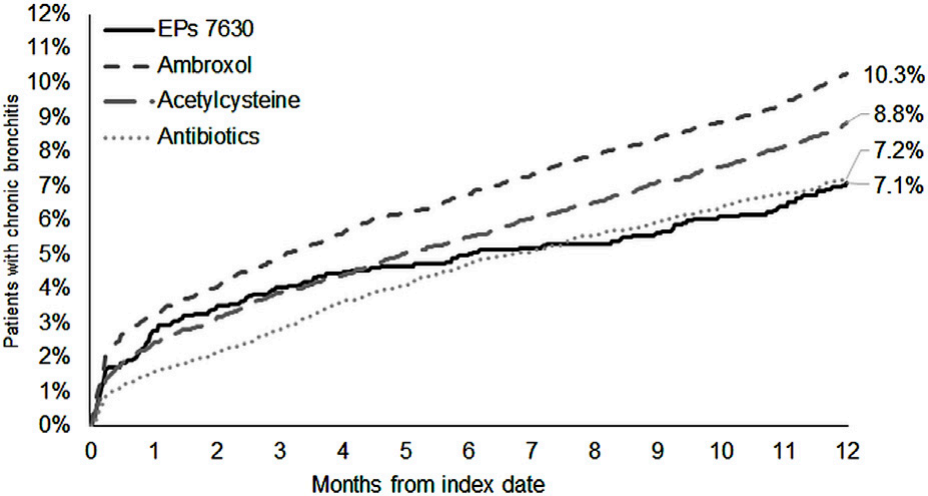
Cumulative incidence of chronic bronchitis in the period 1–365 days after the index date.

The cumulative incidence of chronic bronchitis was lowest in the EPs 7630 cohort (7.1%) compared to the ambroxol (10.3%) and acetylcysteine (8.8%) cohorts (Figure 5). EPs 7630 was significantly associated with a lower risk of chronic bronchitis compared to ambroxol (HR: 0.78; 95% CI: 0.70–0.88) and acetylcysteine (HR: 0.81; 95% CI: 0.72–0.91) (p < 0.001 each; Table 2). No difference was observed between the EPs 7630 cohort and antibiotics cohort. Since the proportional Hazard assumption was not fulfilled, additional logistic regression was performed resulting in an Odds Ratio (OR) of 0.97 (95% CI: 0.85–1.10, p = 0.608) for the incidence of chronic bronchitis in the EPs 7630 cohort compared to the antibiotics cohort.

### 3.4 Incidence of CAP

**FIGURE 6:**
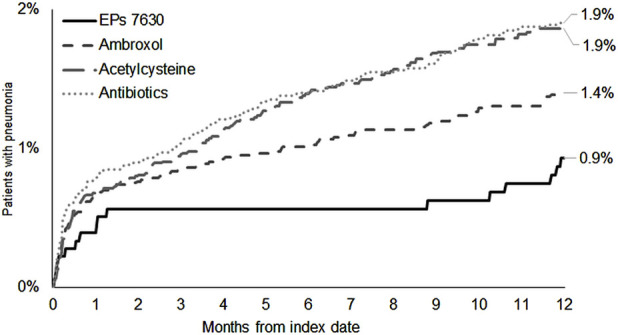
Cumulative incidence of CAP in the period 1–365 days after the index date.

The cumulative incidence of CAP was lowest in the EPs 7630 cohort (0.9%) (Figure 6). EPs 7630 was significantly associated with a lower risk of CAP compared to acetylcysteine (HR: 0.49; 95% CI: 0.29–0.82) and antibiotics (HR: 0.48; 95% CI: 0.29–0.80) (p < 0.003 and p = 0.005, respectively; Table 2). Compared to ambroxol, CAP risk was lower in the EPs 7630 cohort, but the association was not significant (HR: 0.66; 95% CI: 0.39–1.11) (Table 2).

### 3.5 Sick leave associated with acute bronchitis

**FIGURE 7:**
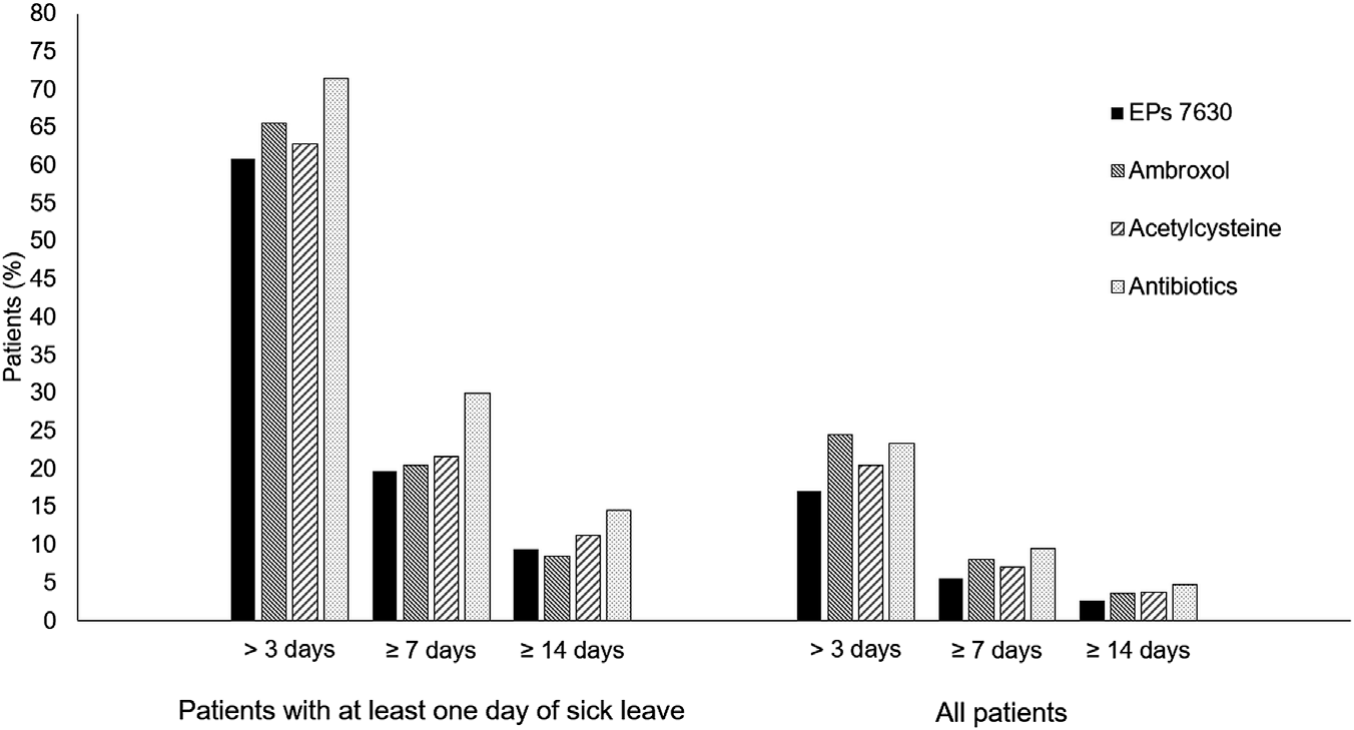
Proportion of patients with sick leave duration of >3, ≥7, and ≥14 days.

Among all patients, the proportion with a sick leave duration of more than 3, at least 7, and at least 14 days was lowest in the EPs 7630 cohort (Figure 7) and prescription of EPs 7630 was associated with a significantly lower risk of sick leave compared to the other groups (for p-values, please refer to Table 4).

**TABLE 4:** Association between EPs 7630 prescription and probability of sick leave of >3, ≥7, and ≥14 days (EPs 7630 versus other therapies).

Sick leave duration	> 3 days		≥ 7 days		≥ 14 days	
	OR (95% CI)	P-value	OR (95% CI)	P-value	OR (95% CI)	P-value
Group of patients with at least 1 day of sick leave						
EPs 7630 vs ambroxol	0.81 (0.66–1.00)	0.047	0.95 (0.74–1.22)	0.699	1.14 (0.80–1.61)	0.469
EPs 7630 vs acetylcysteine	0.95 (0.74–1.14)	0.426	0.89 (0.69–1.16)	0.383	0.83 (0.59–1.18)	0.293
EPs 7630 vs antibiotics	0.62 (0.50–0.77)	<0.001	0.58 (0.45–0.74)	<0.001	0.61 (0.44–0.85)	0.004
All study participants						
EPs 7630 vs ambroxol	0.63 (0.56–0.72)	<0.001	0.68 (0.55–0.73)	<0.001	0.72 (0.54–0.97)	0.030
EPs 7630 vs acetylcysteine	0.80 (0.70–0.91)	<0.001	0.76 (0.72–0.94)	0.011	0.71 (0.53–0.97)	0.022
EPs 7630 vs antibiotics	0.67 (0.59–0.76)	<0.001	0.56 (0.45–0.68)	<0.001	0.55 (0.41–0.74)	<0.001

CI, confidence interval; OR, odds ratio.

Among the group of patients with at least 1 day of sick leave, the risk of sick leave was lower in the EPs 7630 cohort compared to the other cohorts for all sick leave durations, with the exception of the comparison to the ambroxol group for a sick leave of 14 days or more (Table 4). The same applies to the proportion of patients affected (Figure 7). As concerns a sick leave duration of ≥14 days, the proportion of patients was slightly lower in the ambroxol cohort. For durations of >3, ≥7, and ≥14 days in the patient population with at least 1 day of sick leave, the highest proportions of patients were seen in the antibiotics cohort each (Figure 7). This also resulted in a significantly lower risk for sick leave for the EPs 7630 prescription group compared to the antibiotics group (Table 4).

## 4 Discussion

Potential short-term and long-term benefits of prescribing EPs 7630 for managing acute bronchitis can be shown when comparing EPs 7630 to other commonly used treatments such as ambroxol, acetylcysteine, and antibiotics.

Our real-world data analysis indicates a lower incidence of renewed acute bronchitis diagnoses in the EPs 7630 cohort, both within a week and within 1 year. HR for EPs 7630 indicated a 26% to 22% lower risk of recurrence of acute bronchitis compared to the other cohorts. This suggests a potential preventive effect of EPs 7630 against recurrent bronchitis episodes which might be explained by the ability of EPs 7630 to induce an interferon response, which has been shown to be protective against viral infections (Bojkova et al., 2023; Cinatl et al., 2024).

The cumulative incidence of new antibiotic prescriptions was slightly lower in the EPs 7630 cohort compared to the ambroxol and acetylcysteine cohorts. Compared to acetylcysteine, the difference was statistically significant. Notably, the Kaplan-Meier curves did not diverge until after approximately 6 months. This could possibly be explained by the preventive effect or by the cohort differences discussed before. Importantly, after the first month, i.e., presumably after the acute episode of acute bronchitis had subsided, there was a considerable difference in new antibiotic prescriptions between the EPs 7630 cohort and patients who were initially treated with an antibiotic. This difference may reflect the prescribing habits of individual physicians. It was already observed in the same database that physician characteristics are more important drivers of antibiotic prescriptions than the patients themselves (Kern and Kostev, 2021). Overall, looking at the group sizes of the prescription groups before propensity score matching, a high prescription rate for antibiotics is noticeable in our study: monotherapy and co-medication combined, 79% of patients had received antibiotics as a first treatment of their acute bronchitis. In terms of monotherapies only, the percentage was up to 89%. This clearly contrasts with the consistent advice of evidence-based guidelines that antibiotics are rarely indicated for acute bronchitis, which is usually of viral origin (Schubert et al., 2023).

An antibiotic prescription rate clearly higher than the 20% target rate was also found in a study analyzing secondary data from a German insurance company (Arens et al., 2024). In fact, the authors reported an antibiotic prescription in 35% (13,465/38,913) of patients with acute bronchitis. This shows that prescribing rates and inappropriate use of antimicrobials in outpatients seeking care for acute respiratory infections such as acute bronchitis remain high. A survey of 1,000 GPs in the UK revealed that 55% felt under pressure to prescribe antibiotics, even if unsure whether they were necessary, and 44% admitted they had previously prescribed antibiotics to end an appointment and get the patient to leave (Cole, 2014). However, it was shown that patient views about antibiotic treatment were not useful for identifying those who will benefit from antibiotics (Coenen et al., 2013). In a large prospective study conducted in the US primary and urgent care setting, antibiotics showed no measurable impact on the severity or duration of cough caused by acute lower inspiratory tract infection (Merenstein et al., 2024), which emphasizes that in most cases of acute bronchitis an antibiotic treatment is unnecessary and offers no added benefits. Moreover, a retrospective cohort study from Sweden, which analyzed patient data collected between 2014 and 2020, demonstrated that the risk for infectious complications from common upper respiratory tract infections such as acute bronchitis was low and not modified by antibiotic treatment (Dahlén et al., 2022).

The overall trend seen in our study suggests that prescribing EPs 7630 may contribute to a reduced need for, and trigger a lower prescription rate of, antibiotic therapy. Therefore, the use of phytopharmaceuticals such as EPs 7630, which has been shown to have a low risk of side effects (Matthys and Köhler, 2010; Matthys et al., 2013), is a viable alternative while mitigating the risks associated with antibiotics, such as the evolution of antibiotic resistance or side effects. Furthermore, EPs 7630 patients on sick leave generally had a shorter duration of sick leave compared to those on antibiotics. It should be noted that the duration of sick leave cannot be equated with the duration of illness, which is not recorded in the database. Nevertheless, EPs 7630 seems to reduce the loss of workdays due to sick leave, which could potentially limit economic costs. However, cost reduction has not been calculated in this study.

A reduction in antibiotic use through the initial administration of different phytopharmaceuticals for acute bronchitis and other acute respiratory tract infections was also shown by earlier real-world data analysis (Martin et al., 2020; Kassner et al., 2024). While this may somehow indicate a class effect, the aforementioned studies also showed differences in the effects of the different phytopharmaceutical drugs analyzed, particularly with regard to the incidence of recurrent or chronic bronchitis (Kassner et al., 2024) and to the association between the phytopharmaceutical prescription and sick leave (Martin et al., 2020). This is consistent with the fact that the efficacy of herbal compounds depends heavily on the plants used and the extraction and standardization methods employed, which means that the results obtained for one preparation cannot be extrapolated to other preparations using the same plant or combination of plants (Kardos, 2015). This applies to the same or an even greater extent to preparations made from other plants.

The costs of viral respiratory tract infections such as acute bronchitis have been recognized as a major economic burden due to decreased productivity and disease-related inability to work (Birnbaum et al., 2002). The effectiveness of EPs 7630 in reducing the duration of sick leave compared to placebo was shown in various trials. In a meta-analysis of four randomized placebo-controlled trials, 7 days of treatment with EPs 7630 significantly reduced the average number of sick days and significantly increased the proportion of patients who were able to return to work after 1 week (Matthys et al., 2023). However, the fact that antibiotics were prescribed may suggest a higher severity of illness in the antibiotics cohort, which could explain the longer duration of sick leave compared to the EPs 7630 cohort. Furthermore, the prescription of sick leave at the first doctor’s visit due to acute bronchitis does not provide any information on the effectiveness of the prescribed medication, as the sick leave was issued before the medication was taken.

Complications of acute bronchitis are chronic cough, progression to chronic bronchitis, and CAP (Wark, 2015). A prospective study found that, within the first month of the disease, 20% of patients presented again with persistent or recurrent symptoms, mostly with a persistent cough (Macfarlane et al., 2001). Another prospective study showed that, 3 years after initial presentation with acute bronchitis, 34% of patients had symptoms consistent with either chronic bronchitis or asthma (Jónsson et al., 1998). Compared to these data, the incidence of chronic bronchitis was comparable in the ambroxol cohort, lower in the acetylcysteine cohort, and lowest in the EPs 7630 and antibiotics cohorts. This suggests that EPs 7630 and antibiotics may help to prevent the occurrence of chronic bronchitis. Regarding EPs 7630, this might be due to the combination of antiviral and immunomodulatory effects which enable EPs 7630 to counteract the later stages of a viral respiratory infection caused by an overshooting immune response (Cinatl et al., 2024).

The incidence of CAP was below 2% in all cohorts. EPs 7630 prescription was found to be significantly associated with a lower risk of CAP compared to the prescriptions of acetylcysteine and antibiotics, suggesting that this drug might have the potential to prevent CAP as a complication of acute bronchitis. This might reflect antibacterial effects of the compound which are mediated by inhibition of bacterial adhesion to viable epithelial cells and bactericidal and bacteriostatic effects, among others (Cinatl et al., 2024). For example, EPs 7630 was shown to have antibacterial effects against *Staphylococcus aureus*, *Streptococcus pneumoniae*, *beta-hemolytic Streptococcus*, *Escherichia coli*, *Klebsiella pneumonia*, *Proteus mirabilis*, *Pseudomonas aeruginosa* and *Haemophilus influenzae in vitro* (Kolodziej, 2011). In an open-label study, patients with mild to moderate uncomplicated acute bacterial rhinosinusitis were randomly assigned to treatment with either EPs 7630 or amoxicillin (Perić et al., 2020). Samples of discharge were taken from the middle meatus of the patients and cultivated for bacteria. At the end of the treatment period, fewer bacterial types were found in the EPs 7630 group compared to the amoxicillin group. Overall, EPs 7630 thus seems to have the potential to reduce complications associated with acute bronchitis due to its antiviral and antibacterial properties.

### 4.1 Limitations

Retrospective analyses of primary care data are generally limited by the validity and completeness of the data available. In the IQVIA™ Disease Analyzer database, diagnoses rely on the ICD codes entered by the GPs. Those codes do not allow for the separation of viral and bacterial infections or severity stages of the disease or outcome. It should also be noted that patients suffering from asthma or COPD were not excluded from the analysis, and therefore exacerbations of these diseases may have been incorrectly documented as acute bronchitis. However, as the final study cohorts were well matched in terms of diagnosis of asthma or COPD, a comparable risk of misdiagnosis can be assumed, ensuring that the comparison between groups remains meaningful. Moreover, prescription data may not adequately capture actual adherence to medication or usage patterns, neither in relation to the initial prescription nor to the period up to 365 days after the index date. In Germany, physicians can issue a prescription for herbal medicines, but this is not mandatory, as these are over the counter (OTC) drugs whose costs are not covered by statutory health insurance. The IQVIA™ Disease Analyzer database does not include data on the use of herbal medicines (e.g., EPs 7630) or other OTC drugs (e.g., non-steroidal anti-inflammatory drugs) that patients buy freely. Consequently, those pharmaceuticals may be underrepresented in the database. The sustained effect of EPs 7630 over the 365-day period could therefore be the result of repeated independent use by patients who were satisfied with the initial effect of EPs 7630. In contrast, antibiotics require a prescription and may thus be overrepresented. Moreover, a doctor’s visit may suggest that the illness is more severe compared to patients who buy cold medication directly from the pharmacy without prior consultation of a physician which might limit the generalizability of the results to the broader, possibly healthier population.

It should further be noted that, in the IQVIA™ Disease Analyzer database, patients can only be observed in a single practice. Cases where a patient receives another diagnosis or prescription from another physician or hospital are not documented in the patient’s data record. Due to the nature of the database, data on socioeconomic status and lifestyle-related risk factors (smoking, alcohol consumption, physical activity) are not available.

Differences in the recurrence rate of acute bronchitis may also partly reflect differences in patient cohorts not captured in the database and therefore could not be controlled for, such as symptom severity and duration, occupational or social exposure to other infected individuals, or lifestyle.

### 4.2 Conclusion

The prescription of EPs 7630 is associated with a reduction in the need for antibiotic therapy, in the loss of workdays, in the incidence of complications, and in the incidence of disease recurrence. The lower incidence of recurrent acute bronchitis and the reduced risk of chronic bronchitis suggest that EPs 7630 has a potential preventive effect against the long-term effects of acute bronchitis, as well as against recurrent bronchitic episodes. This emphasizes the use of EPs 7630 as an effective treatment option for managing acute bronchitis while mitigating the risks associated with inappropriate antibiotic therapy.

## Data Availability

This study was an analysis of German IQVIA™ Disease Analyzer database data obtained under license from IQVIA Commercial GmbH & Co. OHG. The data related to the study are reported in the paper. The raw data cannot be publicly shared as it was obtained from a third party and as per signed agreement. Requests for data can be sent to IQVIA Commercial GmbH & Co. OHG. and may carry a cost. Further information on purchasing IQVIA™ Disease Analyzer database data can be found at https://www.iqvia.com/de-de/locations/germany. Our study findings can be replicated by obtaining the IQVIA™ Disease Analyzer database data and using the methods detailed in the manuscript. No authors had any special access privileges in obtaining the data that others would not have. Any additional information required to reanalyze the data reported in this paper is available from the corresponding author upon request.
